# Exploring the Biosynthetic Potential of Microorganisms from the South China Sea Cold Seep Using Culture-Dependent and Culture-Independent Approaches

**DOI:** 10.3390/md23080313

**Published:** 2025-07-30

**Authors:** Gang-Ao Hu, Huai-Ying Sun, Qun-Jian Yin, He Wang, Shi-Yi Liu, Bin-Gui Wang, Hong Wang, Xin Li, Bin Wei

**Affiliations:** 1 Zhejiang Key Laboratory of Green, Low-Carbon, and Efficient Development of Marine Fishery Resources, College of Pharmaceutical Science & Collaborative Innovation Center of Yangtze River Delta Region Green Pharmaceuticals, Zhejiang University of Technology, Hangzhou 310014, China; 201706030718@zjut.edu.cn (G.-A.H.); 221122230078@zjut.edu.cn (H.-Y.S.); 202005530209@zjut.edu.cn (H.W.); 201906030311@zjut.edu.cn (S.-Y.L.); hongw@zjut.edu.cn (H.W.); 2 Fourth Institute of Oceanography, Ministry of Natural Resources, Beihai 536000, China; yinyin2026@163.com; 3 CAS and Shandong Province Key Laboratory of Experimental Marine Biology, Institute of Oceanology, Chinese Academy of Sciences, Nanhai Road 7, Qingdao 266071, China; wangbg@qdio.ac.cn; 4 Binjiang Institute of Artificial Intelligence, Zhejiang University of Technology (ZJUT), Hangzhou 310051, China

**Keywords:** cold seeps, 16S rRNA sequencing, metagenomic, biosynthetic potential, secondary metabolite

## Abstract

Cold seep ecosystems harbor unique microbial communities with potential for producing secondary metabolites. However, the metabolic potential of cold seep microorganisms in the South China Sea remains under-recognized. This study employed both culture-dependent and culture-independent approaches, including 16S rRNA amplicon sequencing and metagenomics, to investigate microbial communities and their potential for secondary metabolite production in the South China Sea cold seep. The results indicate microbial composition varied little between two non-reductive sediments but differed significantly from the reductive sediment, primarily due to Planctomycetes and Actinobacteria. Predicting the Secondary Metabolism Potential using Amplicon (PSMPA) predictions revealed 115 strains encoding more than 10 biosynthetic gene clusters (BGCs), with lower BGC abundance in reductive sediment. Culture-dependent studies showed Firmicutes as the dominant cultivable phylum, with strains from shallow samples encoding fewer BGCs. Metagenomic data confirmed distinct microbial compositions and BGC distributions across sediment types, with cold seep type having a stronger influence than geographic location. Certain BGCs showed strong correlations with sediment depth, reflecting microbial adaptation to nutrient-limited environments. This study provides a comprehensive analysis of the metabolic capabilities of South China Sea cold seep microorganisms and reveals key factors influencing their secondary metabolic potential, offering valuable insights for the efficient exploration of cold seep biological resources.

## 1. Introduction

Cold seeps are distinctive environments formed by the release of organic-rich fluids, such as methane, from deep-sea sediments onto the seafloor [1,2]. Characterized by chemosynthesis independent of photosynthesis, cold seeps are among the most remarkable extreme environments in the deep sea [3]. The harsh conditions of low temperature, low oxygen, high pressure, and absence of light exert evolutionary pressures, shaping the unique microbial communities in these ecosystems [4,5]. Among the predominant microbes are anaerobic methane-oxidizing archaea and sulfate-reducing bacteria [6,7], which thrive by metabolizing hydrocarbon-rich fluids, including methane and other short-chain alkanes seeping from the seabed. This methane release supports microbial life and impacts global climate by introducing greenhouse gases into the atmosphere [8]. In addition to well-characterized microbes, cold seep sediments harbor a vast diversity of uncultured microorganisms, including many potentially novel bacterial and archaeal species [9,10,11,12]. Despite their diversity, most of these microbes cannot be cultivated under laboratory conditions due to the extreme nature of their environment and the limitations of current enrichment techniques [13]. To survive in the resource-limited and highly competitive deep-sea environment [14], many of these microorganisms have evolved unique metabolic pathways [15,16], potentially producing bioactive compounds with ecological functions to gain a competitive advantage [3,17,18].

Secondary metabolites are produced by microorganisms through biosynthetic gene clusters (BGCs) as a means of self-defense or environmental adaptation. Collectively, microbes generate a remarkable diversity of secondary metabolites, which differ in their chemical structures, biosynthetic pathways, and physiological functions. Many of these compounds have either been approved for clinical use or are undergoing various stages of clinical trials [19,20,21]. Major classes of secondary metabolites include ribosomally synthesized and post-translationally modified peptides (RiPPs), polyketide synthases (PKSs), nonribosomal peptides (NRPs), and terpenes [22]. As of early 2022, 86 secondary metabolites derived from cold seep organisms had been identified, nearly half of which were novel, and approximately 60% exhibited multiple biological activities [3]. However, our earlier research revealed that at least 96.8% of the secondary metabolic potential in marine prokaryotes remains unexplored, suggesting that a wealth of unknown natural products in cold seep environments awaits discovery [23]. Due to the technical constraints of deep-sea sampling equipment and methodologies, our current understanding of microbial communities in cold seep ecosystems remains incomplete. This is particularly evident in knowledge gaps regarding variations in microbial composition and secondary metabolic potential across different cold seep sites and sediment depths. Furthermore, traditional methods for natural product discovery have proven inadequate, posing a significant bottleneck in the development of deep-sea microbial resources.

In recent years, advances in high-throughput sequencing technologies and computational algorithms have significantly advanced microbiome research [24,25]. This has led to a growing body of metagenomic studies investigating microbial communities across diverse environments, including marine ecosystems [26,27], deep-sea cold seeps [16,28], soils [29], and human microbiomes [30,31]. Yu et al. [28] analyzed 113 marine cold seep metagenomes, yielding 49 million non-redundant genes and 3175 metagenome-assembled genomes (MAGs), along with 1895 operational taxonomic units (OTUs) at the species level. Remarkably, over 90% of these OTUs were identified as novel compared to existing databases, underscoring the immense and largely unexplored microbial diversity. Similarly, Dong et al. [16] analyzed 81 marine cold seep metagenomes, identifying 2479 MAGs, which included multiple uncultivated bacterial and archaeal candidate phyla. Moreover, genome mining has revolutionized natural product discovery by overcoming challenges such as microbial culturing difficulties and the repeated rediscovery of known compounds. Advanced computational tools like antiSMASH [32], DeepBGC [33], and PRISM [34] leverage comparative algorithms and deep learning to efficiently identify BGCs from genomic data, enabling the discovery of novel bioactive compounds. These findings underscore the power of culture-independent approaches in uncovering novel microorganisms and expanding our understanding of their metabolic capacity.

Despite advances in metagenomics, the relationship between microbial community composition and secondary metabolic potential in cold seeps remains poorly understood. To address this knowledge gap, we employed an integrated multi-omics strategy that combines the strengths of both culture-dependent and culture-independent approaches. Through systematic enrichment cultivation from six deep-sea cold seep sediment sites, we isolated 518 bacterial strains, providing valuable cultured resources for functional validation. To complement these efforts, we conducted comprehensive culture-independent analyses, including 16S rRNA amplicon sequencing and metagenomics, to achieve a more thorough assessment of microbial diversity and metabolic potential. A key innovation of our study is the application of our in-house developed PSMPA (Predicting the Secondary Metabolism Potential using Amplicon) [35] platform to comparatively analyze both the bacterial OTUs and isolates, revealing novel insights into secondary metabolite biosynthesis. Furthermore, our metagenomic investigation not only validated the secondary metabolic potential but also uncovered unique biosynthetic novelty and distinctive distribution patterns among South China Sea cold seep microorganisms. This synergistic combination of cultivation and omics approaches provides unprecedented, multi-dimensional understanding of cold seep microbial communities and their metabolic capabilities.

## 2. Results

### 2.1. 16S rRNA Sequencing Reveals the Bacterial Diversity and Metabolic Potential in Cold Seeps

**Figure 1:**
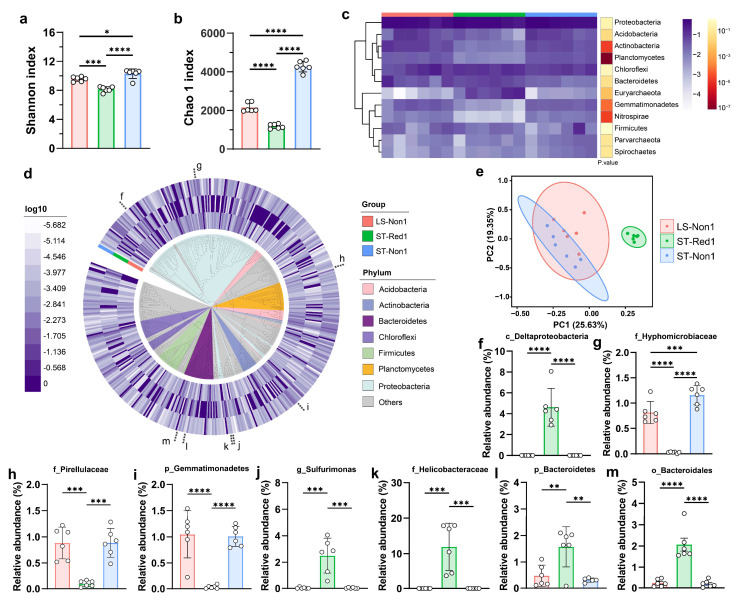
(**a**) Shannon index and (**b**) Chao 1 index of the microbial community in the reductive sediment and two non-reductive sediments from the South China Sea cold seep. (**c**) Microbial composition at the phylum level in the three sampling locations. (**d**) Phylogenetic tree of the 485 OTUs with total abundance > 100. The data are represented in layers from inner to outer: OTU classification at the phylum level and the relative abundance of OTUs across the three sampling locations. (**e**) PCA plot of microbial composition grouped by sampling location. (**f**–**m**) Eight OTUs showing significant inter-group variation. * *p* < 0.05, ** *p* < 0.01, *** *p* < 0.001, and **** *p* < 0.0001.

16S rRNA sequencing was performed on 18 samples collected from different depths across one reductive sediment and two non-reductive sediments at the South China Sea cold seep. A total of 2.7 million raw reads were obtained, with the number of reads per sample ranging from 98,370 to 319,483 (an average of 151,098 reads per sample). Sequencing data were analyzed using QIIME2 [36], resulting in the assembly of 29,560 unique 16S rRNA sequences. The sequencing reads were successfully mapped to 1037 unique OTUs (Table S3). Taxonomic classification revealed that only 100 OTUs (9.6%) could be confidently identified at the species level, while 353 OTUs (34.0%) were classified at the genus level. Significant differences in microbial α-diversity were observed between the samples collected from two types of cold seep. The Shannon and Chao1 indices of the microbial community in the reductive sediment from the southwest Taiwan cold seep were significantly lower than those in the non-reductive sediments, indicating reduced microbial diversity in the reductive environment. This difference was consistent across all replicate samples (*n* = 6 per site), demonstrating robust ecological variation between seep types rather than sampling artifacts. Moreover, the microbial diversity (Shannon index) and richness (Chao 1 index) in the non-reductive sediment from the southwest of Taiwan Island were higher than those in the non-reductive sediment from Lingshui, Hainan Island (Figure 1a,b). The identified OTUs encompassed 73 distinct bacterial phyla, with Proteobacteria (26.1–60.7%), Chloroflexi (4.0–25.7%), and Acidobacteria (1.4–12.4%) emerging as the dominant phyla in these samples. Among the groups, Planctomycetes and Actinobacteria showed the greatest inter-group variability (*p* < 0.0001) (Figure 1c).

Phylogenetic analysis of 485 high-abundance OTUs revealed the evolutionary relationships of these OTUs, as well as the differentially abundant OTUs among the three cold seep sampling sites (Figure 1d). PCA revealed a high degree of microbial compositional similarity between the two non-reductive sediments, while the reductive sediment clustered distinctly along PC1 (Figure 1e). For instance, *c_Deltaproteobacteria*, *g_Sulfurimonas*, *f_Helicobacteraceae*, and *o_Bacteroidales* showed high abundance in the reductive sediment, whereas *f_Hyphomicrobiaceae*, *f_Pirellulaceae*, and *p_Gemmatimonadetes* were more abundant in the two non-reductive sediments (ANOVA, *p* < 0.01) (Figure 1f–m). These findings highlight the ecological uniqueness of cold seeps, where specialized microbial communities coexist with a broad range of uncultured taxa.

**Figure 2:**
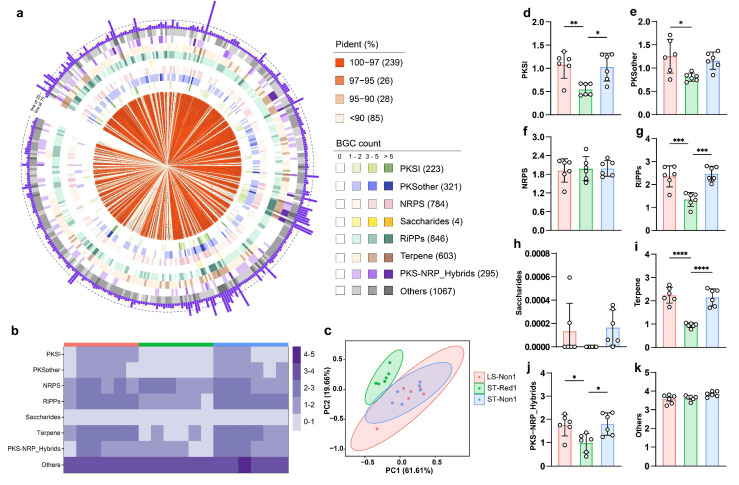
(**a**) Phylogenetic tree of the 378 bacterial OTUs with a total of ≥5 BGCs. The data are represented in layers from inner to outer: 16S rRNA sequence percent identity relative to the PSMPA database, the distribution of various BGC classes encoded by the OTUs, and the total number of BGCs encoded by each ©. (**b**) Heatmap illustrating the distribution of different BGC types across the three sampling locations. (**c**) PCA plot of BGC composition grouped by sampling location. (**d**–**k**) Abundance of eight distinct BGC classes across the three sampling locations. * *p* < 0.05, ** *p* < 0.01 and *** *p* < 0.001, and **** *p* < 0.0001.

PSMPA was used to predict the distribution of secondary metabolite BGCs in sediment samples from three locations in the South China Sea cold seep, based on 16S rRNA amplicons. PSMPA confirmed the presence of novel microbial lineages in these samples, with 613 OTUs (62%) showing <95% similarity and 426 OTUs (43%) exhibiting <90% similarity to reference database entries (Table S4). The phylogenetic tree of 378 bacterial OTUs predicted to possess more than five BGCs revealed that the strains in these samples had the highest biosynthetic potential. Among them, 115 strains encoded more than ten BGCs, with sixty of them belonging to the phylum Actinobacteria (Figure 2a). In total, 4142 BGCs were predicted from these 378 bacterial OTUs. The three most prevalent types were RiPPs (846 BGCs, 20.4%), NRPS (783 BGCs, 18.9%), and terpenes (601 BGCs, 14.5%) (Figure 2b). The distribution of BGCs was strongly concordant with previous findings on the secondary metabolism of cold seep environments [16,28]. Principal component analysis of BGC composition revealed distinct clustering patterns, where the two non-reductive sediments shared highly similar metabolic profiles, while the reductive sediment formed a distinct cluster (Figure 2c). Inter-site heterogeneity analysis revealed distinct BGC distribution patterns: NRPS showed similar abundance across all sites (ANOVA, *p* > 0.05), while the two non-reductive sediments were enriched in other BGC classes (e.g., PKSI: ANOVA, *p* < 0.05; terpene: ANOVA, *p* < 0.0001). Notably, the non-reductive sediment from the southwest of Taiwan Island harbored the most diverse BGC repertoire, aligning with its highest microbial diversity. This highlights the role of habitat-specific selection pressures in shaping microbial composition and biosynthetic potential.

### 2.2. Enrichment and Selective Culturing Yield 518 Bacterial Isolations from Cold Seep

**Figure 3:**
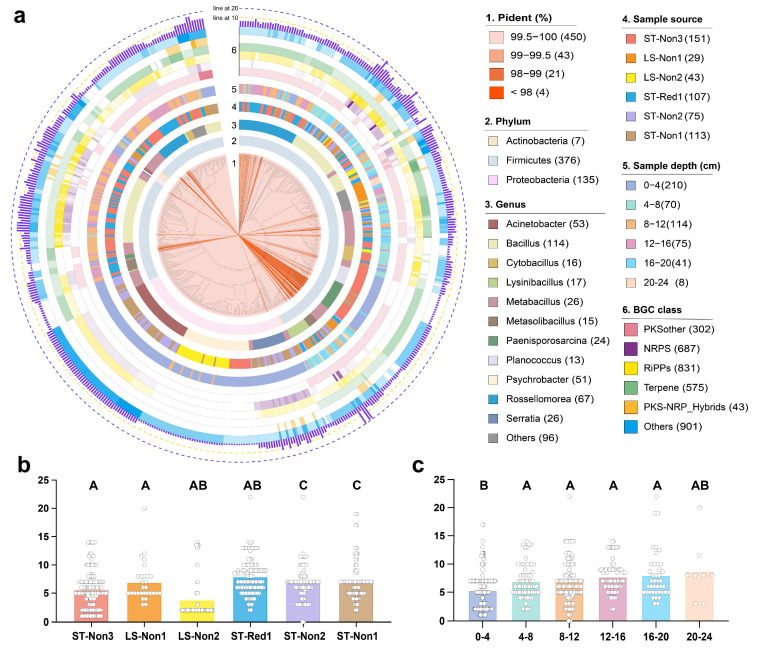
(**a**) Phylogenetic tree of the 518 isolated bacterial strains. The data are represented in layers from inner to outer: 16S rRNA sequence percent identity relative to the PSMPA database, bacterial taxonomy at the phylum level, bacterial taxonomy at the genus level, isolation sample source, isolation sample depth, and the distribution of various BGC classes encoded by the isolates. Bar charts depict the number of BGCs encoded by isolates from (**b**) different sample sources and (**c**) different sample depths. Values with different uppercase letters indicate significant differences (*p* < 0.01).

In this study, microorganisms were isolated and identified from South China Sea cold seep sediment samples collected from six sites, using enrichment and selective culturing methods. A total of 518 bacterial strains were obtained, predominantly classified into Firmicutes (376 strains, 72.6%), Proteobacteria (135 strains, 26.1%), and Actinobacteria (only 7 strains, 1.3%) (Tables S2 and S5). At the genus level, the isolates were classified into 30 genera, with the most abundant being *Bacillus* (Firmicutes), *Rossellomorea* (Firmicutes) and *Acinetobacter* (Proteobacteria), representing 22.0%, 13.0%, and 10.2%, respectively (Figure 3a). Notably, 493 strains (95%) showed over 99% sequence similarity to known 16S rRNA sequences in existing databases. However, certain genera, such as *Metabacillus*, along with some strains from *Paenisporosarcina* and *Rossellomorea*, exhibited low sequence similarity, suggesting the potential presence of novel strains. Microbial distribution showed some site-specific patterns. For example, strains from the genus *Psychrobacter* were predominantly isolated from two non-reductive sediment samples collected from Linshui (Hainan) and the southwest of Taiwan Island at a depth of 0–4 cm, while *Acinetobacter* strains were mainly obtained from two non-reductive sediment samples in the southwest of Taiwan Island at similar depths. All *Planococcus* isolates were originated from a non-reductive sediment sample from the southwest of Taiwan Island at 0–4 cm, whereas *Bacillus* was widely distributed across all six sites (Figure 3a).

A total of 3339 BGCs were predicted from the 518 isolates using PSMPA. Among these, the three most prevalent types were RiPPs, NRPS, and terpenes, accounting for 24.9%, 20.6%, and 17.2% of the total, respectively. This distribution aligns with the results of the 16S rRNA amplicon analysis described above. Significant differences in BGC numbers were observed among genera; for instance, *Bacillus* had the highest average BGC count at 10.2 per strain, followed by *Serratia* with an average of 8.2 BGCs, whereas *Psychrobacter* encoded only 1.7 BGCs on average. The average number of BGCs encoded by the bacterial isolates from each cold seep location varied. Strains from the sole reductive sediment exhibited the highest average BGC count, with 7.9 BGCs per strain, whereas those from the non-reductive sediments at Linshui (Hainan) showed the lowest average of 3.8 BGCs (Figure 3b). Interestingly, in deeper sediment layers, the average number of BGCs per strain increased from 5.3 to 8.7 (Figure 3c). This trend aligns with the findings of Dong et al. [16], who reported significantly higher biosynthetic gene expression in deeper sediments. These results suggest that the biosynthetic potential of cold seep microorganisms is influenced by depth and site.

### 2.3. Metagenomic Study Confirms Microbial Differences Across Cold Seeps

**Figure 4:**
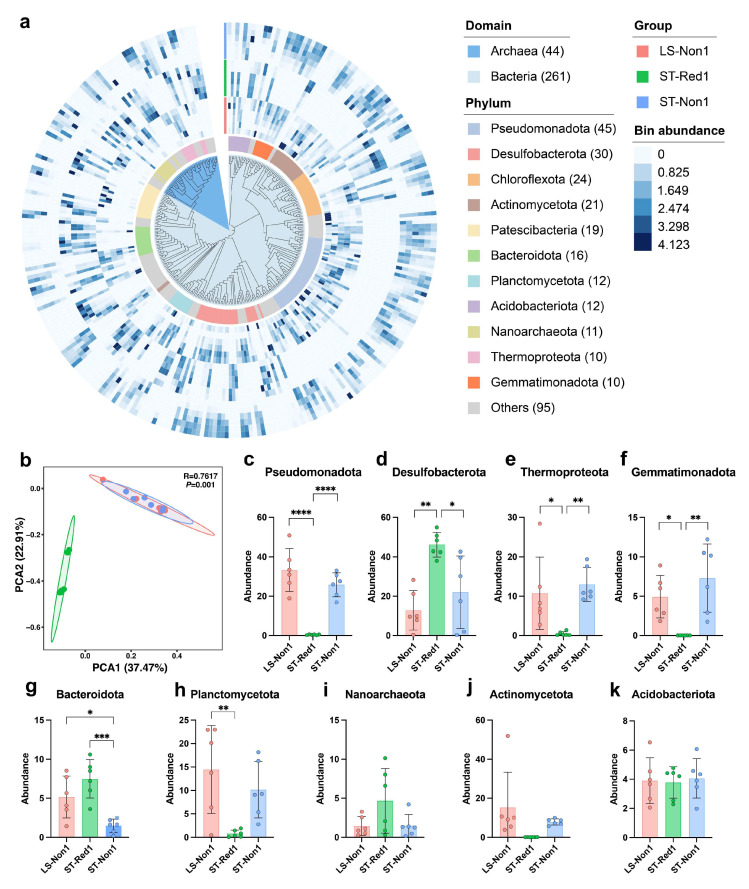
(**a**) Phylogenetic tree of the 305 MAGs. The data are represented in layers from inner to outer: bacterial taxonomy at the domain level, bacterial taxonomy at the phylum level, MAG abundance across all samples. (**b**) PCA of microbial community composition from samples collected at three different cold seep sites. Bar chart depicting the phylum-level abundance of (**c**) Pseudomonadota, (**d**) Desulfobacterota, (**e**) Thermoproteota, (**f**) Gemmatimonadota, (**g**) Bacteroidota, (**h**) Planctomycetota, (**i**) Nanoarchaeota, (**j**) Actinomycetota, and (**k**) Acidobacteriota across the sites. * *p* < 0.05, ** *p* < 0.01 *** *p* < 0.001, and **** *p* < 0.0001.

To further investigate the microbial composition and secondary metabolite potential of cold seep environments, we performed metagenomic sequencing on 18 sediment samples collected from three distinct sites. The assembly and binning yielded 305 medium-to high-quality MAGs, all meeting the Minimum Information about a Metagenome-Assembled Genome (MIMAG) standards (completeness > 50%, contamination < 10%) [37], with an average completeness of 68.2% and a mean contamination of 4.2%. These MAGs exhibited considerable variability in genomic sizes (0.42 Mb to 7.7 Mb) and GC content (27.8% to 68.3%). Taxonomic annotation using the GTDB-tk (release R220) revealed extensive diversity, with 44 MAGs classified as archaea and 261 as bacteria, spanning 47 different phyla (Table S6). Among these, the phylum Pseudomonadota (formerly Proteobacteria) was the most prevalent, accounting for 14.8% of the all MAGs, followed by Desulfobacterota (9.8%) and Chloroflexota (7.8%). Figure 4a presents the phylogenetic tree of all 305 MAGs and their relative abundances across the samples. Our analysis indicates notable differences in community composition between the two cold seep types. PCA results show that samples from the reductive sediment (ST-Red1) clustered separately, with a clear distinction along PC axes (*R* = 0.7617, *p* = 0.001), corroborating previous findings (Figure 4b).

Interestingly, Pseudomonadota were predominantly found in two non-reductive sediments (LS-Non1 and ST-Non1) (Figure 4c), while they were nearly absent in ST-Red1 (ANOVA, *p* < 0.0001). In contrast, Desulfobacterota, Bacteroidota, and Chloroflexota were more abundant in ST-Red1 (Figure 4a,d) (ANOVA, *p* < 0.05), whereas other phyla, such as Thermoproteota, Gemmatimonadota, Planctomycetota, and Actinomycetota were predominantly detected in LS-Non1 and ST-Non1 (Figure 4e–k) (ANOVA, *p* < 0.05). This pattern underscores significant site-specific differences in microbial composition within cold seep environments. Notably, the cold seep site ST-Red1 is characterized by reduced sediments and elevated hydrogen sulfide (H_2_S) concentrations. These high H_2_S levels are likely attributable to the dominant Desulfobacterota strains present in the samples.

### 2.4. Cold Seep Microbes Harbor Diverse and Site-Specific BGCs

**Figure 5:**
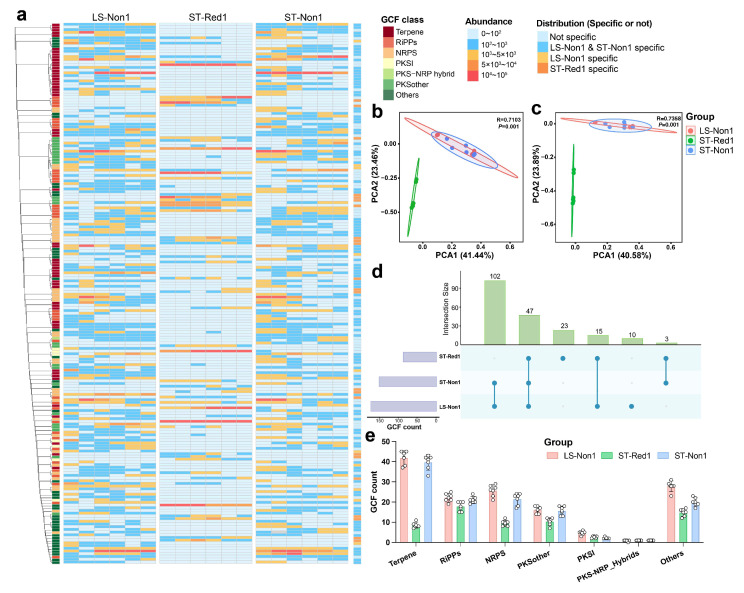
(**a**) Heatmap displaying the total abundance of the top 200 GCFs across the three cold seep sites. (**b**) PCA based on the abundance of the top 200 GCFs across the three cold seep sites. (**c**) PCA based on the abundance of all 910 GCFs across the three cold seep sites. (**d**) Upset plot illustrating the distribution of GCFs across the three cold seep sites. (**e**) Bar chart showing the differential abundance of GCFs.

A total of 928 BGCs were identified from the 305 MAGs using antiSMASH (v7.0.0), with lengths ranging from 1 kb to 52 kb (Tables S7 and S8). These BGCs were classified into seven of the eight BiG-SCAPE classes: NRPS (*n* = 387), terpenes (*n* = 144), RiPPs (*n* = 128), other PKSs (*n* = 76), type I PKSs (*n* = 43), PKS-NRPS hybrids (*n* = 2), and others (*n* = 147). Consistent with previous studies, many incomplete BGCs were recovered at the edge of metagenomic contigs [16,38]. To further assess the biosynthetic potential and diversity of these communities, we employed the Biosynthetic Gene cluster Meta’omics Abundance Profiler (BiG-MAP), a tool designed to profile gene cluster abundance and expression across metagenomic datasets [39]. Under default parameters, the 928 BGCs were clustered into 910 gene cluster families (GCFs), highlighting the high diversity and novelty of BGCs within in the cold seep microbiome. Notably, GCF abundance varied markedly across different sampling sites. Figure 5a illustrates that the top 200 most abundant GCFs are predominantly found in two non-reductive sediments (LS-Non1 and ST-Non1), indicating a greater secondary metabolic potential at these locations. PCA further demonstrated that samples from the reductive sediment (ST-Red1) are distinctly separated from the other sites along both PC1 and PC2 (*p* = 0.001), whether the analysis was based on the top 200 GCFs (Figure 5b) or all 910 GCFs (Figure 5c). Further, Upset plot analysis revealed that LS-Non1and ST-Non1 uniquely shared 102 GCFs, whereas ST-Red1 possessed 23 unique GCFs (Figure 5d). Intriguingly, all 23 of these GCFs were singletons; among them, 8 were attributed to the phylum Desulfobacterota, suggesting that the dominant Desulfobacterota in ST-Red1 encode bioactive compounds providing an adaptive advantage in this unique environment. Among these biosynthetic clusters, seven encoded NRPS and four encoded RiPPs, suggesting a potential survival advantage in competition. This indicates that microorganisms produce antimicrobial peptides to maintain their niche in the challenging cold seep ecosystem. Importantly, these BGCs are highly novel, with no known analogs in Biosynthetic Gene Cluster (MIBiG) database [40]. Moreover, analysis of the top 200 GCFs revealed that terpene BGCs were the most abundant overall. However, their abundance in ST-Red1 was significantly lower than in the two non-reductive sediment sites, LS-Non1 and ST-Non1, a trend similarly observed for NRPS BGCs (Figure 5e).

### 2.5. Depth-Dependent Distribution and Function of Biosynthetic Gene Clusters in Cold Seeps

**Figure 6:**
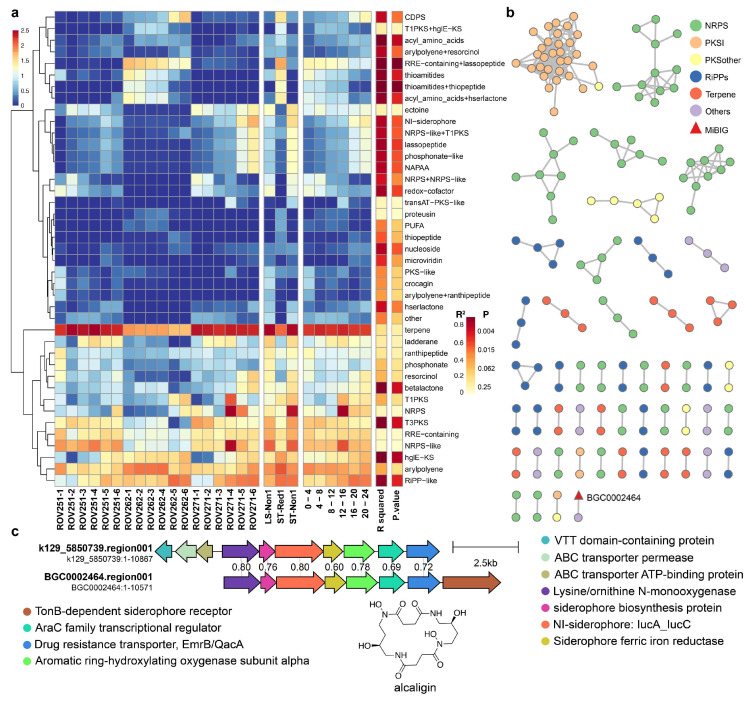
(**a**) Heatmap showing the total abundance of all 42 BGC classes across the three cold seep samples, categorized by sites and depth. (**b**) BGC similarity network of 928 BGCs analyzed using BiG-SCAPE; singletons are not shown, and node colors indicate different BGC types. (**c**) Similarity relationships between hypothetical BGC from cold seep and reference BGCs from the MiBIG database within the identified GCFs.

To investigate the distribution patterns and function of BGCs across different cold seep locations and sediment depths, we performed a comprehensive analysis of BGC abundance across multiple samples. A total of 42 classes of BGCs were identified by antiSMASH. Among the three distinct cold seep sites, the most abundant BGCs belonged to the terpenes, followed by NRPS-like and RiPPs. Notably, the abundance of terpenes was significantly lower in the reductive sediment (ST-Red1) compared to the other two non-reductive sediments (LS-Non1 and ST-Non1) (Figure 6a). Conversely, certain BGC types exhibited higher abundance in ST-Red1, including CDPS, T1PKS + hglE − KS, acyl_amino_acids, RRE − containing + lassopeptide, thioamitides, thioamitides + thiopeptide, and acyl_amino_acids + hserlactone. Interestingly, these BGCs showed a direct correlation with sediment depth. For instance, CDPS (*R^2^* = 0.7669, *p* = 0.0222) and acyl_amino_acids (*R^2^* = 0.9061, *p* = 0.0034) exhibited a negative correlation with depth, whereas RRE − containing + lassopeptide (*R^2^* = 0.942, *p* = 0.0013), thioamitides (*R^2^* = 0.88, *p* = 0.0056), thioamitides + thiopeptide (*R^2^* = 0.9391, *p* = 0.0014), and acyl_amino_acids + hserlactone (*R^2^* = 0.8678, *p* = 0.0069) showed a positive correlation. Additionally, BGCs such as NI − siderophore (*R^2^* = 0.8089, *p* = 0.0147), NRPS − like + T1PKS (*R^2^* = 0.7968, *p* = 0.0167), and lassopeptide (*R^2^* = 0.8442, *p* = 0.0096) were more abundant in the LS-Non1 and ST-Non1, with their abundance positively correlated with sediment depth. Other BGCs, including betalactone, T3PKS, hglE − KS, and RiPP − like, also exhibited similar depth-related patterns. These observations suggest that environmental conditions within the cold seep sediments vary significantly with depths, prompting microorganisms to adapt through the expression of specific BGCs. In deeper sediment layers, microbes may adapt to more extreme conditions by upregulating biosynthetic genes expression, thereby enhancing their survival and metabolic capabilities.

To further assess the novelty and diversity of these BGCs, the BiG-SCAPE [41] tool was used to cluster the 928 identified BGCs into 51 GCFs and 755 singletons (Figure 6b). Using a cutoff parameter of 0.4, we found that only one GCF, comprising two BGCs, was grouped with reference BGCs from the MIBiG database [40]. This demonstrated a remarkable level of diversity and novelty, with nearly 99.9% (805 vs. 806) of these BGCs remaining unexplored in terms of their functional potential. The annotated GCF included an NI − siderophore BGC (BGC0002464), which encodes the macrocyclic dihydroxamate siderophore alcaligin [42]. Under iron-limited conditions, microorganisms synthesize high-affinity iron-chelating siderophores, such as ferrioxamine E, produced by *Streptomyces* spp., and ferrichrome A, produced by *Aspergillus* spp., both of which exhibit remarkably high iron-binding affinity [43,44]. This mechanism is crucial for maintaining iron homeostasis and supporting various metabolic processes in microorganisms [45]. Compared to BGC0002464, the hypothetical BGC lacks the TonB-dependent siderophore receptor gene present in the reference but includes three additional genes upstream: a VTT domain-containing protein, an ABC transporter permease, and an ABC transporter ATP-binding protein. The shared gene similarity between the hypothetical BGC and the reference one is only 60–80%, indicating that the hypothetical BGC may encode a siderophore compound with a similar structure.

## 3. Discussion

Although previous studies have primarily focused on cold seep communities and their metabolic activities, most have relied on metagenomic analyses [14,16,28,46,47]. In this study, we adopted a multi-strategy approach that integrated culture-independent techniques (metagenomic and 16S rRNA amplicon sequencing) and culture-dependent methods (enrichment and selective culturing) to achieve a more comprehensive exploration of microbial communities and their biosynthetic potential in deep-sea cold seep ecosystems of the South China Sea.

16S rRNA amplicon sequencing is more sensitive for detecting low-abundance microorganisms in environmental samples [48]. This provides an advantage over metagenomic sequencing, which often underrepresents rare species due to sequencing depth limitations [49]. Additionally, culture-based methods, while valuable, are constrained by the fact that many microorganisms, especially those with low abundance, are difficult or impossible to cultivate under laboratory conditions [50]. These limitations further restrict our understanding of microbial communities, particularly those dominated by rare taxa. In this study, 1037 unique OTUs were identified from cold seep samples through 16S rRNA amplicon sequencing, with approximately 66% of these remaining unclassified at the genus level due to limitations in existing databases (Figure 1). This finding underscores the vast diversity of microorganisms in cold seep ecosystems that remain unexplored. Furthermore, we observed that cold seep type had a greater impact on microbial secondary metabolism potential than geographic location. For instance, the reductive sediment ST-Red1 exhibited a notably lower secondary metabolism potential compared to the non-reductive sediments LS-Non1 and ST-Non1, despite LS-Non1 being geographically close to ST-Red1. The unique geochemical conditions in ST-Red1 contributed to reduced lower microbial diversity and abundance, resulting in a lower overall secondary metabolic potential (Figure 1). However, bacterial isolation from these sites revealed that strains from ST-Red1 exhibited the highest average number of BGCs per strain (Figure 2), suggesting potential underrepresentation of strains with elevated secondary metabolism capabilities from other sites due to isolation biases.

Metagenomic sequencing deepened our understanding of the microbial communities in cold seep environments, leading to the assembly of 305 MAGs and the identification of 928 BGCs. Significant variations in microbial community composition and BGC diversity were observed across different sampling locations and sediment depths. At non-reductive sediment sites (LS-Non1 and ST-Non1), members of the phylum Pseudomonadota (formerly Proteobacteria) dominated the microbial communities. In contrast, reductive sediment sites (ST-Red1) were predominantly inhabited by Desulfobacterota, a group of anaerobic sulfate-reducing bacteria (SRB) that utilize sulfate as an electron acceptor to produce hydrogen sulfide (H_2_S) [51] (Figure 4). Cold seep environments are characterized by active hydrocarbon seepage, which releases substantial amounts of H_2_S, a critical energy source for chemosynthetic microorganisms in deep-sea ecosystems [52,53]. The sulfide-rich, reductive sediments of ST-Red1 harbored a distinct microbial community, notably enriched with sulfur-cycling bacteria. Specifically, Desulfobacterota (SRB) were highly abundant, consistent with their metabolic adaptation to sulfate-reducing conditions. Furthermore, 16S rRNA gene amplicon sequencing corroborated these findings, revealing a notable enrichment of *c_Deltaproteobacteria* in ST-Red1 (Figure 1f). Syntrophic SRB, primarily composed of Deltaproteobacteria [54], play a crucial role in sulfur cycling through interspecies hydrogen transfer. Additionally, *g_Sulfurimonas* was significantly enriched in this environment (Figure 1j). *Sulfurimonas* species are known as sulfur-oxidizing bacteria (SOB) that thrive at redox interfaces, such as sulfide-rich sediments [53,55]. The distinct microbial community structure in ST-Red1 reflects the environmental specificity of cold seeps. This uniqueness is driven by sulfur cycling, a process mediated by both sulfate-reducing and sulfur-oxidizing bacteria, which governs nutrient cycling [56].

Both bacterial isolation studies and metagenomic analyses revealed that certain BGCs exhibited correlations with sediment depth (Figure 2 and Figure 6). For example, the analysis of the NI-siderophore BGC showed a notable increase in abundance in deeper sediment layers. This suggests that, as iron availability decreases with depth [57], microorganisms may produce more siderophores to scavenge iron from the surrounding environment. Siderophores like ferrioxamine E and ferrichrome A are essential compounds for microbial iron acquisition, especially in deep-sea ecosystems where iron is often a limiting nutrient [58]. The observed positive correlation between siderophore BGC abundance and sediment depth likely reflects an adaptive strategy adopted by microbial communities to cope with iron-limited conditions in deeper sediments. This adaptive mechanism demonstrates the metabolic flexibility of cold seep microorganisms, allowing them to adjust their biosynthetic processes in response to environmental changes [59]. In deeper sediments, where nutrient availability is more restricted, microorganisms may increase the production of siderophores. This strategy helps them meet their iron requirements for essential cellular processes, such as respiration and enzyme function [60].

Our integrated multi-omics approach, combining culture-independent and culture-dependent methods, reveals distinct metabolic potential in cold-seep microbial communities. Notably, the reductive sediment ST-Red1 harbors a unique microbial profile compared to non-reductive cold seeps. However, current assessments of secondary metabolic potential rely solely on in silico predictions. To fully characterize these biosynthetic capabilities, further experimental validation may be required. Future work may involve non-targeted metabolomics for detecting sediment metabolites, as well as isolation and structural characterization of compounds encoded by novel BGCs.

## 4. Materials and Methods

### 4.1. Cold Seep Sample Source and Collection

A total of 37 cold seep sediment samples were collected from Lingshui and Southwest of Taiwan Island deep-sea cold seep in the South China Sea during 2020 and 2021 (Table S1). Notably, the sampling site ROV262-ST-Red1 exhibited highly reductive conditions, characterized by elevated concentrations of H_2_S in its sediments. The samples were retrieved using push cores deployed by a remotely operated vehicle (ROV) at depths ranging from 0 to 0.26 m below the seafloor. Immediately after collection, the sediments were stored in anaerobic biobags at −80 °C to preserve microbial integrity for future analyses.

### 4.2. Isolation and Culture of Strains

Both routine and enrichment cultivation methods were employed to isolate and cultivate microbial strains from cold seep samples. For enrichment cultivation, sediment samples were inoculated into three different liquid media: Difco Marine Broth 2216 [61], R2A medium, and ISP Medium No.3 (ISP3). Each culture was incubated at 10 °C and 20 °C for 16 h to encourage the growth of diverse microbial communities. In contrast, for routine cultivation, samples were directly added to phosphate-buffered saline (PBS) solution without additional nutrients. From each 5 mL sample-containing solution, 100 µL was withdrawn for gradient dilution. The 10^−3^ and 10^−4^ dilutions were plated onto solid media, including Difco Marine Broth 2216, R2A medium, ISP3, and Actinomycete-selective media supplemented with 25 mg/L nystatin and 25 mg/L nalidixic acid to inhibit fungal contamination [62,63]. Plates were incubated at 10 °C, 20 °C, and 28 °C for colony development. All plating experiments were performed in triplicate to ensure reproducibility. Individual colonies were streaked twice for purification before being transferred to liquid media for further cultivation. The purified bacterial isolates underwent 16S rRNA sequencing and were preserved at −80 °C in nutrient broth supplemented with 20% (*v*/*v*) glycerol for long-term preservation. Comprehensive metadata, including culture conditions, isolation protocols, and additional strain-specific details, are provided in Table S2.

### 4.3. DNA Extraction, Amplification, and 16S rRNA Sequencing

Microbial genomic DNA was extracted from 18 sediment samples (including one reductive sediment site from ST and two non-reductive sediment sites from ST and LS) using the TIANamp Soil DNA Kit (Tiangen Biotech, Beijing, China). The V3–V4 region of the 16S rRNA gene was amplified using universal primers 338F (5′-ACTCCTACGGGAGGCAGCA-3′) and 806R (5′-GGACTACHVGGGTWTCTAAT-3′). Amplicons were purified using Ampure XP beads (A63881, Beckman, CA, USA) and quantified with a Qubit 3.0 fluorometer (Q32854, Invitrogen, CA, USA) using the Qubit dsDNA HS Assay Kit. Sequencing was performed on the Illumina NovaSeq platform using the PE250 protocol by Hangzhou Kaitai Biotechnology Co., Ltd. (Hangzhou, China).

The raw 16S rRNA sequences were processed and analyzed using QIIME2 [36] (version 2024.10). Taxonomic classification was performed using Greengenes2 (2022.10) as the reference database for OTU assignments [64]. The multiple sequence alignment of 16S rRNA gene sequences was conducted with Kalign [65] (v3.4.0), followed by phylogenetic tree construction using FastTree [66] (v2.1.11). The resulting tree was visualized and enhanced using iTOL [67] (Interactive Tree of Life) for a more comprehensive display.

### 4.4. Metagenomic Sequencing, MAG Construction, and Bioinformatics Analysis

For metagenomic sequencing, genomic libraries were prepared using the AT4107-Enzymic Universal DNAseq Library Prep Kit (KAITAI-Bio, AT4107) and sequenced on the Illumina NovaSeq platform (Hangzhou Kaitai Biotechnology Co., Ltd.) with a 150 bp paired-end model. Sequencing generated a total of 273 GB of data, with an average of 15.2 GB per sample. Standard quality control protocols were applied to the raw reads using fastp [68] (v0.23.4) to ensure high-quality data. Assembly of the metagenomes was performed with MEGAHIT [69] (v1.2.9) using a k-mer list ranging from 29 to 129, retaining contigs longer than 1000 bp.

The assembled contigs were binned into MAGs using MetaWRAP [70] (v1.2.1), which integrates results from MetaBAT2, MaxBin2, and CONCOCT for optimal binning performance. MAG refinement was conducted with MetaWRAP’s bin_refinement module, retaining only those MAGs with a completeness greater than 50% and contamination lower than 10%. Taxonomic classification of MAGs was carried out using the Genome Taxonomy Database Toolkit [71] (GTDB, release R220). Phylogenomic analysis was performed using UBCG [72] (Universal Bacterial Core Genes), and the resulting phylogenetic tree was visualized with iTOL.

All genomes were processed using the command-line version of antiSMASH [32] (v7.0.0) with the bacterial setting and default parameters. The number and composition of each class of BGCs in these genomes were extracted from the HTML output files generated by antiSMASH using a custom Python script (available at https://github.com/BioGavin/wlabkit (Accessed on 30 July 2025)). The abundance of BGCs across the different samples was analyzed using the default parameters of BiG-MAP [39]. BGC clustering analysis and networking were performed with BiG-SCAPE [41], applying a cutoff value of 0.4. To compare and visualize the similarity of gene clusters, Clinker [73] was utilized.

### 4.5. Statistical Analysis

Data processing and visualization were carried out using GraphPad Prism 9.0 software and R (v3.6). Simple linear regression analyses were performed in GraphPad Prism 9.0. The networks were visualized using Cytoscape (v3.10.2) [74], and one-way ANOVA was used to determine statistical significance between multiple groups.

## 5. Conclusions

In this study, we employed both culture-dependent and culture-independent approaches to investigate the microbial communities of deep-sea cold seep ecosystems in the South China Sea and assess their potential for production of secondary metabolites. Our analyses revealed that variations in microbial community composition and BGC diversity were more strongly influenced by cold seep type than by specific seep locations. Certain BGCs exhibited strong linear correlations with sediment depth, potentially reflecting the survival mechanisms of cold seep microorganisms in nutrient-limited environments. Metagenomic analyses confirmed distinct microbial community structures and BGC distributions across different sediment types, highlighting the remarkable diversity and distinct distribution patterns of BGCs in the cold seep environment. This study provides a comprehensive analysis of the metabolic capabilities of microorganisms in South China Sea cold seeps, identifying key factors that shape their capacity for secondary metabolite biosynthesis. These findings provide valuable insights for the targeted exploration of cold seep biological resources.

## Data Availability

The metagenomic sequencing data have been deposited in the NCBI database under BioProject ID PRJNA1226410. All 16S rRNA amplicon sequencing data are available in the Zenodo repository under DOI https://doi.org/10.5281/zenodo.14903435. All datasets are publicly accessible and available for download.

## Supplementary Materials

The following supporting information can be downloaded at https://www.mdpi.com/article/10.3390/md23080313/s1: Table S1. Information about Cold Seep Samples; Table S2. Isolation protocols about the 518 isolations; Table S3. 1037 unique OTUs analyzed from Qiime2; Table S4. BGC distribution of 989 Bacterial OTUs; Table S5. BGC distribution of 518 bacterial isolations; Table S6. 305 MAGs abundance in 18 cold seep samples; Table S7. Genomic features of the 305 cold seep MAGs; Table S8. Information about the 928 BGCs.
